# Transitioning from pediatric to adult healthcare with an inborn error of immunity: a qualitative study of the lived experience of youths and their families

**DOI:** 10.3389/fimmu.2023.1211524

**Published:** 2023-07-31

**Authors:** François Ouimet, Justine Fortin, Aline Bogossian, Nicole Padley, Hugo Chapdelaine, Eric Racine

**Affiliations:** ^1^ Pragmatic Health Ethics Research Unit, Institut de recherches cliniques de Montréal, Montréal, QC, Canada; ^2^ École de travail social, Faculté des arts et des sciences, Université de Montréal, Montréal, QC, Canada; ^3^ Faculté de médecine, Université de Montréal, Montréal, QC, Canada; ^4^ Département de médecine et Département de médecine sociale et préventive, Université de Montréal, Montréal, QC, Canada; ^5^ Division of Experimental Medicine, McGill University, Montréal, QC, Canada; ^6^ Department of Neurology and Neurosurgery, McGill University, Montréal, QC, Canada

**Keywords:** transition, inborn errors of immunity, primary immunodeficiencies, pediatric, ethics, chronic illness

## Abstract

**Introduction:**

Transition from pediatric to adult healthcare is a multifaceted and consequential process with important health implications for youth. Although research on transition has grown significantly, research on transition for patients living with an inborn error of immunity (IEI) is scarce. We undertook a qualitative study to better understand the perspectives of youths and parents in an outpatient immunology clinic.

**Methdos:**

Semi-structured interviews were conducted with 9 youths, 6 parents and 5 clinicians, all recruited from the same clinic. All youths recently transferred to adult care with or without an established diagnosis of IEI. Interviews were transcribed verbatim and thematic analysis was conducted. Two sets of themes were generated. The first set captured the positive and negative aspects experienced during transition, as well as recommendations to facilitate the process. The second set focused on key topics discussed in the interviews that were merged into overarching themes.

**Results:**

Perspectives of participants were clustered into 6 overarching themes: (1) lack of knowledge about IEIs; (2) scattered transitions; (3) changing healthcare teams; (4) approaching an unknown environment; (5) transitioning to adulthood; (6) assuming responsibility for the management of the condition. Overall, the challenges encountered with respect to these themes had profound clinical and humanistic implications for patients such as generating significant distress.

**Discussion:**

We discuss the unique challenges of the youths in our study in comparison to common problems reported by youths with chronic illness in the broader transition literature (for example: the change of healthcare team, the lack of information about the transition process and navigating the adult care system, growth towards self-management and the co-occurring developmental transition to adulthood). There is an urgency to attend to the specific problems created by the rarity of IEIs and related lack of knowledge about them as well as the need for multidisciplinary cross-clinic care during transition and beyond.

## 1 Introduction

As they approach adulthood, youths with chronic conditions such as inborn errors of immunity, aka as primary immunodeficiencies, must prepare for a challenging period during which they will transfer from pediatric to adult care. Challenges pertaining to continuity of care, relational continuity, lack of information, and self-management of the condition are now well recognized issues in pediatric-to-adult care transition for chronic conditions (1, 2, Padley N, Moubayed D, Lanteigne A, Ouimet F, Clermont M-J, Fournier A, et al. Transition from pediatric to adult health services: aspirations and practices of human flourishing. Int J Qual Stud Health Well-being (under review - 2023)). Furthermore, youths are susceptible to higher morbidity and mortality during the transition period due to lack of follow-up and poor treatment adherence (3). In addition to medical transition, youths and their families navigate other concurrent transitions in their lives – i.e., the transition from adolescence to adulthood – representing a series of physical, psychological, social, and environmental changes.

Although healthcare transition is now a burgeoning field of research, little attention has been paid to IEIs. This is of concern as each chronic condition presents different treatment requirements and clinical manifestations that may not be accommodated in a homogeneous transition process. For example, IEIs present clinical implications – e.g., the need for highly specialized cross-clinic care and psychosocial and cognitive complications – that may pose additional barriers to a smooth transition. To our knowledge, there are few studies dedicated to healthcare transition in IEIs (3–7), of which only one study has included the perspectives of youths or their families (7). The inclusion of perspectives of patients and their families in research is essential to influence the development of programs or procedures that could respond more specifically to their needs. Losing sight of their perspectives may result in transition programs that are overly centered on treatment adherence and medical compliance, priorities which might not align with the needs and values of youths and their families, leading to suboptimal outcomes (8).

We undertook a qualitative study to better understand the perspectives of youths and parents in an outpatient care immunology clinic dedicated to IEIs. We sought to glean a broader understanding of their experience, their needs, and the challenges they face with regard to the transition from pediatric to adult care.

## 2 Materials and methods

### 2.1 Study aims

We aimed to understand the healthcare transition for youths with IEIs. From the perspectives and the experiences of youths, their caregivers and clinicians we aimed to identify: 1) clinical, ethical, and psychosocial issues that occur during transition for youths with IEIs and with other chronic conditions; 2) strategies and recommendations that could better address these issues within transition programs.

### 2.2 Ethics approval

Ethics approval from the Research Ethics committee of the Institut de recherches cliniques de Montréal (IRCM) was sought and granted (approval no. 2018-941).

We used a focused ethnography methodology in order to study the process of transition for the youths, their caregivers and clinicians, how it operates, and how it affects the different parties, both favorably and unfavorably. A focused ethnography – in contrast to standard ethnography – narrows the scope of the study to a specific context, involving a smaller number of participants and a shorter timeframe (9, 10). This provides an optimal framework to answer the research questions we aim to address, through the study of the institutional norms, structures and practices (i.e., the context), in addition to the experiences of youths, their caregivers, and clinicians.

### 2.3 Participants and recruitment procedure

Participants were recruited at an outpatient adult immunology clinic where clinical nurses served as liaisons between the research team and potential participants. Given that IEIs are frequently undiagnosed (e.g., diagnostic odyssey) and that the immunology clinic sees patients with other conditions, we opted for a generous recruitment strategy to include patients not yet diagnosed. The nurses introduced the study prior to, or following, clinical appointments, and responded to questions. A research team member (JF) was then contacted to meet the potential participants and completed the consent process with participants. Participants were recruited following convenience sampling (11).

Interview participants consisted of three groups: 1) youths aged between 18 and 30, diagnosed or not with an IEI, and recently transferred from the pediatric care center to the adult immunology clinic; 2) caregivers accompanying interviewed patients and; 3) healthcare professionals (clinical nurses, immunologist, administrative officer, genetic counselor) working at the adult immunology clinic.

### 2.4 Interview process

Semi-structured individual interviews were conducted in person or over the phone, audio-recorded and transcribed verbatim by a professional transcription service. Interviews generally lasted between 20 to 60 minutes. Interviews were conducted immediately after the clinic appointment or at another time convenient for the youth and/or caregiver. As for the healthcare professionals, interviews were conducted at the adult immunology clinic in their office at their convenience.

### 2.5 Interview guide

The initial interview guide was developed by two team members of the research team. This guide was then continually adapted throughout the data collection by the authors (JF & AB) who were conducting the interviews.

### 2.6 Coding and data analysis

Data analysis followed thematic content analysis (12, 13). Coding was implemented with the qualitative analysis software MaxQDA. Two phases of coding were conducted with two different sets of codes following the method proposed by Braun and Clarke. This method proposes 6 steps: 1) familiarizing yourself with the data; 2) generating initial codes; 3) searching for themes; 4) reviewing themes; 5) defining and naming themes; 6) producing the report (12).

The first phase of coding was derived deductively and aimed to group excerpts pertaining to the general experience of the transition. These experiences fell under four themes: 1) perceived differences between pediatric and adult care systems 2) positive aspects of the transition 3) negative aspects of the transition and 4) recommendations. Phase 1 themes were applied to interview segments where participants explicitly referred to “differences”, “positives”, “negatives”, or “recommendations”, and to answers following a question explicitly probing on these themes. In the second phase of coding, themes were derived inductively. This second phase aimed to identify topics relating to participants’ experience of the transition process or their experience living with an IEI more generally.

An initial coding guide for phase 1 and 2 was devised by the first author (FO) and revised by the senior author (ER). Phase 2 themes were adjusted iteratively throughout the coding process. A total of 13 codes were used in the final coding stage. Coding was then revised and validated by another co-author (NP) to ensure data triangulation. Once coding was complete, the 13 codes were grouped into 6 overarching themes.

Translation of cited excerpts of interviews was undertaken by a fully bilingual team member (NP) and validated by another bilingual member (FO). Participants are anonymized and labeled correspondingly to their status – Y, CG, HCP for youths, caregivers, and healthcare professionals – and the order their first citation appears in the result section.

## 3 Results

### 3.1 Participant information

A total of 20 interviews were conducted with 9 youths, 6 caregivers and 5 healthcare professionals. All 9 youths had recently transferred to adult care. The mean age of youths was 21.4 years old. Of the 9 youths interviewed, four were diagnosed with an IEI, one with chronic granulomatous disease, one with a pediatric autoimmune neuropsychiatric disorder associated with streptococcal infections (PANDAS), one with velocardial facial syndrome and two were still waiting on a diagnosis.

### 3.2 Experience of transition

**Figure 1:**
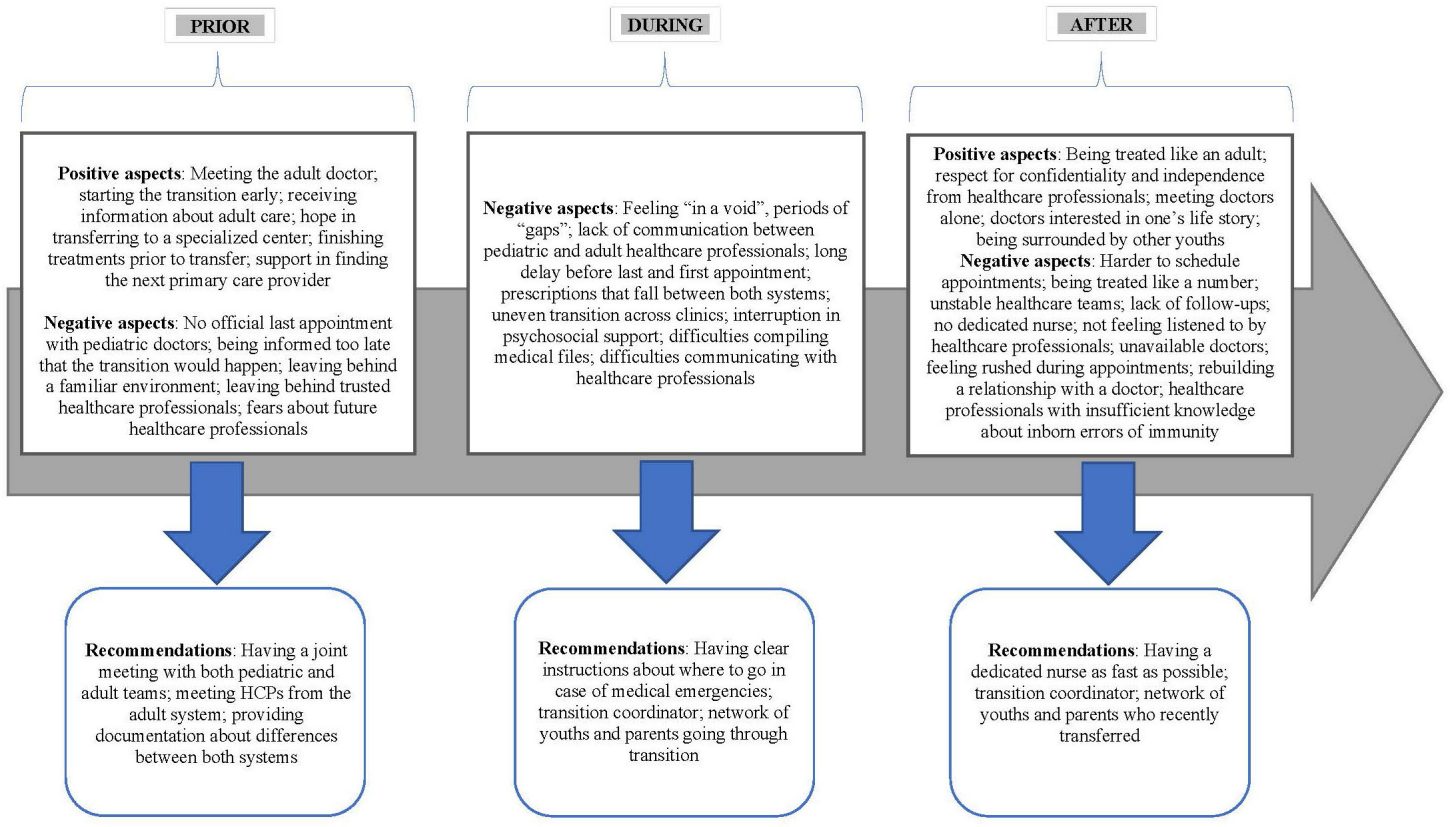
Experience of transition. Positive aspects, negative aspects and recommendations reported by youths and their families.

Participants were asked directly about the main differences they perceived between pediatric and adult healthcare systems, the positive and negative aspects they experienced during their transition, and their recommendations to facilitate the transition process. Answers were grouped into four themes “Perceived differences”^1^, “Reported positive aspects”, “Reported difficulties” and “Recommendations” (See **Figure 1**).

### 3.3 Transitioning to adult care with an IEI

A total of six overarching themes were formed from the 13 codes applied during phase 2 of coding. Theme 1 (“Lack of knowledge about IEIs”) and theme 2 (“Scattered transitions”) emerged as particularly important as they represent challenges more specific to IEIs. Theme 3 (“Changing healthcare teams”) also emerged as a key issue in interviews. Three other themes were also formed: theme 4 (“Approaching an unknown environment”), theme 5 (“Transitioning to adulthood”) and theme 6 (“Assuming responsibility in the management of the condition”).

#### 3.3.1 Theme 1: lack of knowledge about IEIs

Lack of knowledge about IEIs was a common theme explicitly or implicitly brought up by participants which encapsulated many challenges that youths and their families faced living daily with condition and transitioning to adult care.

Many participants shared their experience of a long and sometimes painful period before receiving a proper diagnosis and, consequently, before receiving adequate treatment for their condition. This led to negative experiences with the health care system, which had a significant impact – physically and psychologically – on some youths and their families. As this caregiver explained, her son’s first hospitalization caused significant trauma as healthcare professionals struggled to find adequate treatments:

“The doctors really delayed prescribing medication to him because they stayed there just to observe him. They had observed him for two months without giving him anything except some kind of painkillers and that’s it. But not to cure, it’s just to control, calm, but not to cure. The disease kept developing during that time. [ … ] I think it left him with a lot of sequelae.” (CG-1)

This youth waited 7 years before receiving a diagnosis for a PANDAS (Pediatric Autoimmune Neuropsychiatric Disorders Associated with Streptococcal Infections which is a rare disease but not an IEI) diagnosis – after being previously diagnosed with obsessive compulsive disorder – which led to a feeling of discouragement when she learned that most of her previous treatment efforts were made in vain:

“For sure, it was like a little bit of an upheaval to know that basically I didn’t have the right diagnosis and that all the therapies that I had done before and the medication and everything, it’s kind of like I did them for nothing.” (Y-1)

The lack of medical knowledge about IEIs also comes with a scarcity of healthcare professionals that are properly trained to care for patients living with an IEI. When approaching the transition, some participants were worried about finding a new doctor with sufficient understanding of their youths’ condition:

“[ … ] it kind of threw me for a loop when the coordinator told me … Because I said, ‘Do you have any doctors to suggest afterwards?’ She said, ‘No, now you’re falling into a *pfft*.’ When I told her ‘Doctors who know about velocardiofacial syndrome’ she said, ‘Good luck.” So that’s when I went ‘oh boy. Oh boy, okay. [ … ] That’s worrisome.’” (CG-2)

Furthermore, the limited research and the scarcity of reliable information available on IEIs proved to be a barrier to participant’s’ health literacy and health-related capacity building. One caregiver notably recounted her difficulty finding resources on the velocardiofacial syndrome (VCFS) in adults: “But that too, the studies on this syndrome, I have books, I even ordered a book from the United States, it is until adulthood. But once it concerns adulthood, there’s nothing left.” (CG-2)

Limited research and awareness on IEIs also had an impact on participants’ daily life. As this caregiver explained, both patients and their caregivers and have a hard time being understood by doctors and acquaintances: “…when my daughter is sick, no matter what I say, no one understands what I’m saying, it’s like I’m speaking another language! It’s fun to be in places where people understand what you’re going through. You can go consult anywhere for cancer, but for PANDAS, you can’t go anywhere.” (CG-3)

#### 3.3.2 Theme 2: scattered transitions

Most patients received care from a variety of clinics (e.g., allergy, pneumatology, gastroenterology, dermatology) in the pediatric system. Consequently, when moving to adult healthcare, many participants experienced a “scattered” or uncoordinated transition across different clinics which seems to be a prevalent challenge when transitioning patients living with IEI, as this healthcare professional explains:

“It’s that in cases where there are several specialists, each one has their own way of doing the transition. So sometimes, it gets complicated. Then each one has its age for when they do the transition, each one has its preferred site. Like, pediatric cardiology at [pediatric hospital] sends to the [adult care center]. So, the poor patient, he finds himself a bit all over the place, no transition is all the time at the same time, that’s something that’s difficult. Sometimes, there are things that fall between two chairs, and then it often falls on our shoulders here in immuno, because I think we are the most stable transition team at the moment.” (HCP-1)

Uncoordinated transitions were a source of great concern for many youths, who felt “thrown into the adult world” and “in the void” as they waited for follow-ups from different clinics. As this caregiver – whose youth experienced interruptions of care in different clinics – explained:

“It’s hard and it’s a lot of stress for the youth with the medication. We had a family doctor who took charge of her, so it was the family doctor who did the renewals of specialists’ medication, but that’s not ideal either. So, it’s a lot of questions about who is going to do the follow-up, how. It’s a lot of unknowns too.” (CG-4)

Ideally, this caregiver said, it would be best “to know to whom you’re going, and not be on waiting lists waiting until they call you. Like meeting [the adult immunologist] at [pediatric hospital], I think, that was reassuring for [my youth].” (CG-4)

Additionally, the change from receiving multidisciplinary care in one center to receiving care from different clinics in different centers was a new challenge for many patients living with IEI. As this healthcare professional noted, a big advantage of pediatric care for patients living with IEI is the integrated multidisciplinary care they receive: “Really on the administrative side, that’s the advantage of [pediatric hospital], it’s a referral hospital where they have everything *in-house*, whereas here in adults, everything is a bit scattered.” (HCP-1)

Such complex multi-clinic transitions complicated communication between healthcare providers in the adult system, as this healthcare professional admitted: “Unfortunately, communication between institutions is not very good.” (HCP-1). This lack of communication was a perceived problem for many youths and caregivers and led to problematic situations where patients received inadequate care because of incomplete medical files. As this youth explained, this had an impact on their trust towards healthcare professionals and sense of security: “Yeah, you know I don’t feel super secure because they forget. Not that they forget, it’s normal to forget, but you know, that they don’t read everything.” (Y-2)

Although some participants admitted that their medical files were “large” and “tough to read”, and appreciated healthcare professionals who were sympathetic to their situation, they were still left feeling uncared for because of incidents where healthcare professionals were not well informed about their medical history, as this caregiver explains:

“It’s like him that was doing the briefing of his own case, without them reading the file, because his file is so thick, maybe I understand those who don’t read. It’s so big. There were some who sympathized with him, who understood him very well. Since then, they try to understand, to help him, to the best of their ability. On the other hand, there were some who were really insensitive, as if they were dealing with a number, not a person, not a human being. It’s just like a number in the hospital, that’s all.” (CG-1)

#### 3.3.3 Theme 3: changing healthcare teams

Changing healthcare teams was reported as one of the key challenges of healthcare transition, notably in terms of breaking ties with the pediatric care team and difficulties with the attitudes and approaches of healthcare professionals in the adult system.

Leaving behind the pediatric care team can be a devastating process for youths who have often known these care providers over many years and through many critical life episodes. As this caregiver explained, her youth’s close bond with the pediatric doctor made for a particularly difficult separation:

“You also get attached when you’ve been with the same team for eighteen years. So much that [my youth], his allergist when she was young, she wanted to marry him. She loved him so much! So, we went to see him, we went to disturb him to say goodbye. And both were quite sad. He was the one who found and relieved her of her eczema, who “saved her from her eczema”. That’s what bothered her the most. So, it was emotional for her.” (CG-5)

Meeting the adult doctor before the transition and, ideally, meeting them with the pediatric doctor present, was frequently recommended as a way to facilitate this process. As this caregiver explained, a meeting with both doctors could help prevent unnecessary fears and doubts while waiting for their first appointment with the adult care doctor:

“I’m just telling you that we would have felt … Less nervous. Because when they tell us: “You’re going to meet [adult immunologist].” But who is he? Are we going to feel comfortable? Will we like him? Will we feel understood? We’ve been in the void for eighteen years.” (CG-5)

Participants also encountered many difficulties when encountering adult healthcare professionals for the first time. They described adult healthcare professionals as “less attentive”, “less understanding”, more “in a rush”. Participants did not “feel welcomed” and often felt like they were treated “like a number”. As this caregiver expressed, healthcare professionals in adult care did not adapt their approach to accommodate youth:

“Once in adult care, he was considered a full-fledged adult, whereas he could not be called that, a young adult, not like a real adult. They imposed certain things on him that he was against, he was a teenager, you shouldn’t rush a teenager.” (CG-1)

Furthermore, many participants reported that they did not feel listened to by adult doctors. This led to inadequate care and problematic interactions, as patients with long-term conditions are often experts of their condition, but nevertheless, can be misinformed about medication or other aspects of their condition (e.g., Dilaudid does not thin the blood as suggested in this example):

“I know my situation; I’ve been living with myself for 21 years. I came in with a mouth infection. They wanted to give me something because I couldn’t walk too much, I had a big arthritis crisis, they gave me fentanyl, morphine, Dilaudid. Dilaudid thins the blood. I have a blood factor that is complicated, you can’t give me a blood thinner. If I fall, I cut myself or whatever, I bleed out. So, I tell them. He says, “Well anyway, that’s not what we were ordered to do.” I said, “Fine, but I’m not taking your pill. You can write in the file that I refuse it.” So you know, you have to fight constantly, constantly. Like I tell them I’m allergic to latex. “Ah it’s not a big deal.” They put on latex gloves. It’s because I become swollen all over. You know your situation, but they’re not listening to you at all.” (Y-3)

#### 3.3.4 Theme 4: approaching an unknown environment

Many participants reported that they had not received enough information about the transition process and the adult care system before leaving pediatric care. The transition was described as a leap into the unknown where uncertainty was part of the process. Participants reported that they were given vague information, and fully realized the differences between both systems only when receiving care in adult centers for the first time: “[ … ] they just said, they had insinuated there was really a difference between pediatric and adult hospitals. [ … ] But that’s all they said. It was once we got to the hospital that we understood, my son too, he really understood what the difference was.” (CG-1)

Consequently, many participants experienced unpleasant and unexpected changes when being treated in adult centers. These experiences ranged from general differences in philosophy of care to specific details about how adult hospitals function. For example, this caregiver said that she and her youth “didn’t know that once at the adult hospital, that he [her youth] wasn’t going to have a [hospital] room for himself [ … ] We didn’t know, I don’t think even my husband knew.” (CG-1)

This was a very distressing situation as her youth, being immunocompromised, always had an individual room in pediatric care. Further, sharing a room with others resulted in fears and feelings of vulnerability. This caregiver blamed this negligence for the fact that her youth ended up in intensive care three times during his hospitalization.

Some participants did not know that the transition occurred at 18 years old and thought that “in certain cases [the pediatric] hospital kept the child until 21 years old” (CG-6). As this caregiver further explained: “It’s weird, we knew 18 was the adult age, but we thought since our daughter has a mental age of seven, eight or whatever, that they would keep her for her exceptional conditions. But no.” (CG-6)

One participant even reported not knowing that her youth would eventually be transferred somewhere else, saying that when she learned about the imminent transition, she was “I was panicking about who is going to follow her and how it’s going to work.” (CG-5)

#### 3.3.5 Theme 5: transitioning to adulthood

The healthcare transition coincides with an important developmental stage in youths’ lives: the passage from adolescence into adulthood. Patients living with IEI reported challenges related to their daily lives and growth towards independence. As this youth explained, the responsibilities of adult life compounded with the management of her condition:

“When I was young, I used to say, “I can’t wait to be 18 and an adult”, but what a shitty idea I had. [ … ] You know, when you’re an adult, you have bills to pay, you work to pay your bills, to pay for food and then rent. I’m working right now to pay for medicine, my rent, my food. What do I have left? 25 dollars a week. It’s a shame. [ … ] That’s basic survival. Right now, that’s the only thing I’m doing, surviving.” (Y-3)

For many participants, their condition had a significant impact on their ability to work, attend school and pursue meaningful hobbies. These challenges were notably posed by the cognitive problems associated with IEIs that require special education and pose certain limits to integrating the workplace, but also by recurrent hospitalizations, lack of energy and the burden of managing their condition. Almost all participants had to miss extended periods of school for treatments or recovery. Furthermore, the development of their condition sometimes required the abandonment of hobbies they had pursued for a long time, as this youth explained: “I was a sporty girl in life. I used to cheerlead, and then when I was diagnosed with juvenile polyarthritis, I had to quit cheerleading and training a lot.” (Y-3) Youths faced additional challenges when bosses and colleagues lacked empathy and understanding: “I had another job three years ago and they just didn’t understand. I asked for a leave of absence for the hospital, and they fired me because it was too much for them.” (Y-3)

#### 3.3.6 Theme 6: assuming responsibility for the management of the condition

As with many health conditions that create complex medical needs, caregivers of patients living with IEI often have a significant role in the management of their youths’ condition. Caregivers’ involvement differed, but their roles commonly included speaking to doctors during appointments, managing appointments, assisting in administrating treatments, logistical support (e.g., transport, coordination with school), and emotional support.

As youths approach adulthood and transition to adult care, they anticipated greater independence in their care and health management. This theme was notably discussed with respect to parents’ decreasing involvement as their child matured into adulthood. Participants expressed variable attitudes towards this expectation of greater independence and responsibility. For instance, many youths liked meeting their doctors alone, where they felt more “in control” and preferred being asked questions directly: “If I ever have any concerns no matter what, I know my mom is there, but I much rather be asked how *I* feel, if this medication has helped *me*.” (Y-4) Some even expressed that they outgrew the approach of pediatric care and felt it was time for a change: “At some point at the end, I was starting to think it was unimpressive that they were still calling my mom to give the results and stuff. I was like, “It’s my record, I’m over 14.” At some point, it’s going to be something that I wouldn’t want her to know, and I don’t feel like always giving them to my mom, the results. They [my parents] are so used to it being like that…” (Y-1) Others were simply indifferent about their caregivers’ involvement: “whether they’re there or not, it doesn’t really bother me”. (Y-5)

Generally, caregivers had a more difficult time accepting their change in role in their youth’s health affairs ‘notably, because of the time invested and sacrifices they may made throughout the years to ensure their child’s health. As this caregiver explained: “I stopped working because there were too many hospitalizations and I – every mother is different – and I … it is not she who chose to come into the world with an illness, so I will never leave her alone for an appointment, no matter how old she is, or for a hospitalization”. (CG-5)

## 4 Discussion

In this paper, we report the results of one of the rare investigations on healthcare transition in the context of IEIs. To our knowledge, no qualitative study has been done to investigate the experience of pediatric to adult healthcare transition of patients living with IEI and their families These patients present with highly varied clinical features, therefore, there are considerable differences in the challenges they face in the transition to adult care (4). Our results reveal that youths with diagnosed or undiagnosed IEIs face similar challenges to youths with other complex medical needs, particularly with respect to the change of healthcare team, the lack of information about the transition and adult care, the transition to adulthood, and expectations of self-management in adulthood (1, 2, Padley N, Moubayed D, Lanteigne A, Ouimet F, Clermont M-J, Fournier A, et al. Transition from pediatric to adult health services: aspirations and practices of human flourishing. Int J Qual Stud Health Well-being (under review - 2023)). These youths also face unique challenges pertaining to the rarity of IEIs and the multidisciplinary care they require. The current study shows the remarkable human impact of these challenges. We first discuss the common challenges that chronically ill youths face during transition then discuss the issues that are more specific to the clinical and psychosocial features of IEIs and related recommendations (**Table 1**).

**Table 1:** Recommendations for clinicians pertaining to transition difficulties*.

Common transition difficulties for chronically ill youths	Recommendations
*Changing of healthcare team*	• Organize joint meetings with pediatric and adult doctor, at pediatric hospital before transfer of establishment (1, 2, 14, 15)
*Approaching an unknown environment*	• Write comprehensive transition plan^2^ with goals and a timeline, 3 years before time of transfer, and update this plan at least once a year (2) • Involve actively patients in elaboration of transition plan (2, 16–18) • Provide information about the differences between pediatric and adult care before transfer (19) • Encourage patients to visit the adult care center before transfer (19)
*Developing into adulthood*	• Adopt a holistic (developmental, biological, clinical and psychosocial) approach to transition (2, 20–23)
*Taking charge in the (co)management of the condition*	• See patients alone before the transfer of centers (2, 18, 24) • Prepare patients at least a year before transfer (18, 24) • Use readiness assessments such as the TRAQ or Transition-Q; integrate these within transition planning and review annually and track progress (2) • Involve all stakeholders (youths, caregivers, clinicians, coordinators) as early as possible in the transition process (25) • Align transfer with developmental and clinical readiness, and not only age (25)
Key transition difficulties for IEI patients	
*Living with a rare medical condition*	• Guide patients and families in their search for new doctors • Time transition to avoid coinciding need for acute medical care examination and investigation • Espouse a pedagogic approach with recently transferred patients • Provide accessible and reliable information sources on IEIs to patients and families • Implement network of peers for patients with similar IEIs to foster information and experience sharing
*Multidisciplinary care and transition*	• Rely on transition coordinators (2, 14, 15, 51, 55) • Provide patients with portable health summaries (e.g., Good 2 Go MyHealth Passport, Portable Patient history, My Health Passport) • Make access easy for: formulated transition plan, one or many transition readiness assessments, and a comprehensive medical summary (2) • Ensure patients have a “medical home” (e.g., community-based family physician supporting coordination between specialist clinicians and acting as a safeguard against discontinuity of care) (2, 56)

*These recommendations are taken from existing literature on transition. However, IEI-specific recommendations (second section of the table) are also grounded in our results.

^2^A comprehensive transition plan should provide a holistic approach to patients needs and include dates, services, health education goals, goals for social leisure, finances, living arrangement, sexual health and assessment of risk behaviors, identification of adult services and providers; goals for social leisure (2). One possibility is for the pediatric team to prepare a summary validated with the patient prior to transition.

### 4.1 Common transition difficulties for chronically ill youths

#### 4.1.1 Change of healthcare team

The change of healthcare team in pediatric-to-adult healthcare transitions is challenging and impactful for patients living with IEI and their caregivers as this change implies losing access to long-term, trusting and secure relationships with members of the pediatric care team, and worry and uncertainty about unknown adult healthcare professionals. Across the transition literature, changing healthcare teams has been reported as one of the main challenges of transition (26, 27), if not the most difficult challenge that youths and their families must face (1). The current study revealed that an important buffer to these challenges is the approach of the adult care team. This approach can be boiled down to four factors: 1) youths and their caregivers met the pediatric and adult doctors at the pediatric center prior to transition, which facilitated a transfer of trust, and prevented a period of uncertainty and fears about the future primary care provider 2) the clinic is small, and staff retention is high, offering a certain stability to recently transferred patients 3) the immunologist provides logistical support by actively taking on responsibilities of care coordination 4) and, the general attitude and qualities of the immunologist, who, contrasted with other adult doctors from which participants received care, was described as compassionate, understanding, patient, open, informative and accessible. The impact that one or few healthcare professionals can have on the general transition experience was evident in our result, and is in line with previous research that illustrate how positive experiences with one good clinician can translate into positive perception about care services in general (28). These positive perceptions can have long lasting effects such as recent evidence that demonstrates increased self-management skills are acquired by youths who receive care in contexts where relational qualities such as empathy and compassion are privileged (29).

#### 4.1.2 Developing into adulthood

The challenges faced by youths living with chronic illness when approaching adulthood have been well documented, and include restrictions on life choices as a result of social and environmental barriers (18, 30, 31). These challenges are compounded for youths with IEIs who report lower social functioning on health-related quality of life scales compared to other chronically ill youths (32, 33). Similarly, youths involved in our study expressed difficulties receiving uninterrupted education, finding, and keeping a job, and pursuing meaningful hobbies, but also, managing the social and existential aspects of the passage to adulthood. Although this topic was not explicitly discussed in our results, one notable challenge for youths with IEIs are the genetic complications of their conditions, and their influence on decisions related to sexual life and their desire to have children. Patients living with IEI also face specific challenges pertaining to the precarity of their immune system, ranging from constraints on career choices (i.e., not working in a virus-friendly environment like a kindergarten) to general limitations on autonomy (i.e., avoiding communal transport). These findings are worrisome as social connections are already a significant challenge for chronically ill youths when compared to their healthy peers (32, 34). Apart from the precarity of their immune system, the rarity of their diseases could also explain why youths with IEIs might be more vulnerable to experiencing social isolation and alienation. Positive relationships are a key dimension of well-being and can provide much-needed support during periods of hardships, as healthcare transition often is. Living with a rare disease makes it harder to connect with peers who face similar challenges and experience the world the same way (26).

#### 4.1.3 Taking charge in the (co)management of the condition

As they approach adulthood, youths with chronic illness are also expected to assume greater responsibility in the management of their condition. This has received significant attention in transition research (35) and is often construed as a “functional” challenge pertaining to youths’ autonomy, health literacy and capacity building. However, taking charge of one’s health and care equally entails reconfiguring the caregivers’ role in the management and support of their youths’ health affairs. This is therefore as much a relational challenge as it is a functional challenge. The reconfiguration of the caregiver-youth relationship is typical of adolescence but is particularly complex in the context of chronic illness such as IEIs since caregivers are often highly invested in their youths’ life and have difficulty taking distances (36, 37). Our results illustrated that perspectives on these shifting roles vary from case to case. In our study on IEIs, some caregivers seemed reluctant to step back when prompted by youths or by healthcare professionals. This is understandable given the bond between youths with chronic illness and their caregivers. However, it may also become an issue if parental involvement infringes upon youths’ desires for confidentiality, privacy and independence which are constitutive of their well-being (38).

Even if youths’ independence should be facilitated and encouraged by healthcare professionals and transition programs in general, autonomy is not an absolute good in itself (39). While acknowledging that parental involvement and readiness for transition are often inversely correlated (40), it is important to recognize that some youths might prefer that their caregivers stay involved under certain parameters they are to determine themselves (41). This is particularly important in the case of IEIs because of possible barriers to health literacy – notably, neurodevelopmental and neuropsychiatric complications and comorbidities and the scarcity of reliable and accessible information on certain IEIs. Autonomy should be conceptualized in terms of empowerment, which relates not only to the acquisition of competences necessary for self-care (e.g., health literacy, capacity-building), but also to the meaning ascribed to those competences and the goals for which they are developed (42). If transition programs are to reflect the priorities of youths, then autonomy should be understood as a relational component – as a form of empowerment – which recognizes the transactional and dynamic dimensions of capacity building (39, 43–45).

#### 4.1.4 Approaching an unknown environment

A pressing issue reported extensively in our results and in many other studies is the lack of information on the transition process and the adult care system (2, 41). As stated in our results, participants experienced anxiety and fears because of the uncertainty surrounding the transition process. Environmental mastery is a crucial dimension of well-being during transition and can be greatly affected by the lack of information about one’s environment, as it restricts one’s sense of control over one’s surroundings (46, Padley N, Moubayed D, Lanteigne A, Ouimet F, Clermont M-J, Fournier A, et al. Transition from pediatric to adult health services: aspirations and practices of human flourishing. Int J Qual Stud Health Well-being (under review - 2023)). Since the transition for patients living with IEIs often involves multiple clinics and sometimes multiple care centers, providing accurate and timely information about the transition process and the intricacies of the adult care system is a particular challenge, as every clinic and care center manages the transition process differently. A case may be made for greater standardization of the transition process across clinics and care centers as the lack of coordination exacerbates youths’ and caregivers’ uncertainty.

### 4.2 Key difficulties for transitioning patients living with IEIs

#### 4.2.1 Living with a rare medical condition

Many patients living with IEI must go through a diagnostic and treatment odyssey before receiving a proper diagnosis for their condition, leading to distress and diminished quality of life (47, 48). As our results show, this odyssey can lead to traumatizing experiences navigating the healthcare system, leaving physiological and psychological scars on youths and their families, which can translate into significant trust issues towards healthcare professionals and the healthcare system in general. This is highly problematic in the context of healthcare transition as the transition itself evokes considerable apprehension about the healthcare system (49, Padley N, Moubayed D, Lanteigne A, Ouimet F, Clermont M-J, Fournier A, et al. Transition from pediatric to adult health services: aspirations and practices of human flourishing. Int J Qual Stud Health Well-being (under review - 2023)). It has been recommended by physicians that patients living with IEI do not transition before receiving a diagnosis (4). While this is a sound recommendation it is concretely infeasible considering the proportion of undiagnosed patients. Our study illustrated that transitioning to a specialized, small-scale, and research-oriented adult clinic holds benefits. These clinics can offer a positive step towards diagnosis and, in the end, prove more beneficial to patients’ well-being than staying in a pediatric setting if it delays diagnosis and proper treatment. This transpired in our results, as one participant with an undiagnosed IEI explicitly stated that she felt renewed hope knowing she will be cared for by a specialized and research-oriented doctor.

As for most rare diseases, experts on IEIs are very few, and healthcare professionals are often unfamiliar with the peculiarities of these disorders (50). This poses challenges to transition in many ways, as youths and their families encounter healthcare professionals with very little knowledge about their condition during a period where their trust in healthcare professionals and the healthcare system in general might be fragile. For example, one participant was shocked to learn that her new family doctor did not even know her disease (self-reported as Di George syndrome) existed. As our results show, families that are not supported enough during the transition process in their search for new doctors can experience fears and anxieties. This lack of general knowledge about IEIs within the medical community is not simply a matter of perception and fears: it can concretely lead to inadequate care during critical periods for youths’ health.

Furthermore, rare diseases pose informational challenges to youths and families themselves, as the lack of accessible resources and general knowledge on IEIs make it much harder for them to grow into experts of their condition. Empowerment through self-mastery and knowledge about one’s own health is of critical value during the transition where young patients are expected to take on more responsibilities in the management of their health affairs (43, 51). Preparing young patients for the new responsibilities expected from them in adult care is already a challenge for many chronic conditions (52–54). A survey on transition for youths with Chronic Granulomatous Disease showed that only 24% of patients are considered to have “disease understanding” (7).

The rarity of IEIs also poses significant challenges outside of the healthcare system, notably, because these disorders are unknown to the general public. Most IEIs do not benefit from the same “public empathy” that better-known diseases such as cancer or diabetes do. Chronically ill youths generally experience more problems socializing and connecting with peers, which can lead to isolation and alienation (34). As stated earlier, connecting with peers who go through similar experiences – friends, classmates, other caregivers or hospital acquaintances – can provide a much-needed emotional and even practical support during the hardships of the disease (26, Padley N, Moubayed D, Lanteigne A, Ouimet F, Clermont M-J, Fournier A, et al. Transition from pediatric to adult health services: aspirations and practices of human flourishing. Int J Qual Stud Health Well-being (under review - 2023)). These are all crucial considerations for healthcare transition which is a period of uprooting from a familiar, comfortable, and trustworthy environment, where psychosocial support is often provided.

#### 4.2.2 Multidisciplinary care and transition

Youths with IEIs and their families faced additional complications during transition due to the coordination of multi-clinic care. Two main problems were identified: an “uncoordinated and “scattered” transition, and problematic interactions with healthcare professionals who lacked information about their patient’s medical history.

Youths with chronic conditions face difficulties adjusting to the logistical challenges that they are expected to take on when transitioning to adult care (e.g., taking appointments, getting to appointments on their own, finding their way across new hospitals).

As healthcare professionals often have limited knowledge about IEIs, efficient communication between healthcare professionals across care systems and across clinics should be a priority to ensure patients living with IEI are well treated. Communication between healthcare professionals – especially between pediatric and adult care – is already a challenge for many transitioning patients with more common chronic illnesses (41, Padley N, Moubayed D, Lanteigne A, Ouimet F, Clermont M-J, Fournier A, et al. Transition from pediatric to adult health services: aspirations and practices of human flourishing. Int J Qual Stud Health Well-being (under review - 2023)) Unsurprisingly, this issue seemed to be recurrent in youths with IEIs, who reported dealing with healthcare professionals who had incomplete knowledge about their medical history and the different information they perceived as important to take into consideration when treating them – wether clinical features, psychosocial aspects, or information about their experience within the healthcare system. This led to situations where patients had to “battle” with healthcare professionals to receive appropriate treatments as their opinion was sometimes discredited.

### 4.3 Strengths and limitations

This study has important limitations. The sample size is small, and all participants were recruited from the same clinic. Furthermore, the experience of interviewed participants might be influenced by the small size of the clinic included in the study, alleviating the likely greater challenges that transitioning patients often face in larger care centers. Additionally, some participants had very limited experience in the adult system as they were transferred very recently, but this project helped gather their apprehensions early in the transition which is a meaningful part of their experience.

Apart from addressing the aforementioned limitations, further research should focus on the psychosocial aspects of transition with IEIs as they were not discussed in-depth in our interviews, but our results, and the general knowledge on IEIs point to significant challenges in that area. Furthermore, there is a lack of quantitative data on transition with IEIs, which could be addressed with surveys including youths and families, but also, longitudinal studies measuring loss to follow-ups across clinics. Additionally, like many other chronic conditions, research on transition in patients living with IEI lack rigorous data on the efficacy of tools such as readiness assessment questionnaires, portable patient history and care coordinators. Finally, although studying every IEI individually is infeasible, groups of IEIs with similar clinical features should be made and investigated together, as there is high variability across the 300 and more IEIs and grouping them together leads to generalizability limitations.

## 5 Conclusion

Transition from pediatric to adult care is a challenge for any chronically ill individual, but youths with IEIs face additional challenges when compared with other chronic conditions. The rarity of IEIs and the multi-clinic care they require pose barriers to youths’ autonomy and continuity of care. Moreover, given the frailty of many patients and their difficulties accessing proper care, challenges associated to transition bear significant health and human implications for them as shown in this study. Significant efforts should be made to empower youths with IEIs and maximize their health literacy as they often have to advocate for their needs when interacting with healthcare professionals unknowledgeable about their diseases in the adult care system. Patients living with IEI would greatly benefit from comprehensive logistical support during transition – e.g., care coordinators and transition clinics – because of the multi-clinic care they require. The small size of the clinic included, and the general attitude of the primary care provider involved in this study seemed to be positive factors for transitioning patients living with IEI.

## Data availability statement

The original contributions presented in the study are included in the article/supplementary materials. Further inquiries can be directed to the corresponding author.

## Ethics statement

The studies involving human participants were reviewed and approved by Research Ethics Committee, IRCM. The patients/participants provided their written informed consent to participate in this study.

## Author contributions

FO: methodology, validation, investigation, data curation, writing - original draft, writing - review & editing, visualization. JF: conceptualization, methodology, writing - review & editing. AB: conceptualization, methodology, validation, writing - review & editing. NP: methodology, formal analysis, data curation, validation, writing - review & editing. HC: supervision, project administration, writing - review & editing. ER: conceptualization, methodology, validation, writing - original draft, writing - review & editing, visualization, supervision, project administration, funding acquisition. All authors contributed to the article and approved the submitted version.

## Footnotes

1 “Perceived differences” is not included in **Figure 1** as the coded excerpts are redundant with “Reported positive aspects” and “Reported difficulties”.
